# Circulating monocyte subsets in multiple myeloma patients receiving autologous stem cell transplantation – a study of the preconditioning status and the course until posttransplant reconstitution for a consecutive group of patients

**DOI:** 10.1186/s12865-019-0323-y

**Published:** 2019-11-08

**Authors:** Ida Marie Rundgren, Elisabeth Ersvær, Aymen Bushra Ahmed, Anita Ryningen, Øystein Bruserud

**Affiliations:** 1 Department of Biomedical Laboratory Scientist Education and Chemical Engineering Faculty of Engineering and Natural Sciences, Western Norway University of Applied Sciences, Bergen, Norway; 2 Department of Clinical Science, University of Bergen, Bergen, Norway; 3 Section for Hematology, Department of Medicine, Haukeland University Hospital, N-5021 Bergen, Norway

**Keywords:** Monocytes, Multiple myeloma, Autologous stem cell transplantation

## Abstract

**Background:**

Induction therapy of multiple myeloma patients prior to autologous stem cell transplantation has changed from conventional chemotherapy to treatment based on proteasome inhibitors or immunomodulatory drugs. We used flow cytometry to analyze total monocyte and monocyte subset (classical, intermediate and non-classical monocytes) peripheral blood levels before and following auto-transplantation for a consecutive group of myeloma patients who had received the presently used induction therapy.

**Results:**

The patients showed normal total monocyte concentrations after induction/stem cell mobilization, but the concentrations of classical monocytes were increased compared with healthy controls. Melphalan conditioning reduced the levels of total CD14^+^ as well as classical and non-classical monocytes, whereas intermediate monocytes were not affected. Thus, melphalan has a non-random effect on monocyte subsets. Melphalan had a stronger effect on total and classical monocyte concentrations for those patients who had received induction therapy including immunomodulatory drugs. Total monocytes and monocyte subset concentrations decreased during the period of pancytopenia, but monocyte reconstitution occurred before hematopoietic reconstitution. However, the fractions of various monocyte subsets varied considerably between patients.

**Conclusions:**

The total level of circulating monocytes is normalized early after auto-transplantation for multiple myeloma, but pre- and post-transplant levels of various monocyte subsets show considerable variation between patients.

## Background

Multiple myeloma is usually characterized by proliferation of abnormal plasma cells in the bone marrow and secretion of monoclonal immunoglobulin [1–3]. Autologous stem cell transplantation is an established part of early myeloma-stabilizing treatment [4, 5], and the patients usually develop a quantitative posttransplant CD4^+^ T cell defect that lasts for several months [6]. The posttransplant innate immune system is less well characterized, but early reconstitution of monocytes with reduced expression of HLA-DR and CD16 together with reduced cytokine production has been observed [7, 8], especially decreased release of proinflammatory cytokines (e.g. IL-6, TNF-α and IL-1β) [8].

Monocytes constitute up to 10% of total circulating peripheral blood leukocytes in healthy individuals [9]; they can differentiate into macrophages or dendritic cells [10] and may also differentiate in endothelial direction [11]. Furthermore, immunomodulatory drugs (IMiDs, e.g. lenalidomide) can induce differentiation towards dendritic cells with modulation of the cytokine profile, the transcriptional regulation and the accessory cell functions [12]. Finally, based on the expression of CD14 (a cell surface co-receptor for lipopolysaccharide) and CD16 (the low affinity IgG receptor) monocytes are now divided into classical (CD14^bright^ CD16^negative^), intermediate (CD14^bright^ CD16^dim^) and non-classical (CD14^dim^ CD16^bright^) monocytes [13–15]. Classical monocytes constitute 90% of the circulating monocytes in healthy individuals [13, 15, 16].

Monocytes seem to be involved in the development of myeloma bone disease [17–20] through the release of soluble mediators that stimulate osteoclastogenesis, and the presence of non-classical monocytes may be a potential marker for increased osteoclast precursors [18, 19]. However, monocytes are also important immunoregulatory cells, and they are important for the defense against complicating infections in myeloma patients [21–23]. Several new drugs have become available during the last decade for the treatment of multiple myeloma, and no previous studies have investigated the effects of these drugs on the levels of circulating monocyte subsets before and following auto-transplantation. In the present study, we therefore used a highly standardized methodology to characterize peripheral blood levels of monocyte subsets in auto-transplanted myeloma patients receiving pre-transplant induction treatment based on proteasome inhibitors and IMiDs.

## Results

### Myeloma patients show decreased concentrations of circulating total leukocytes prior to high-dose melphalan conditioning

**Fig. 1:**
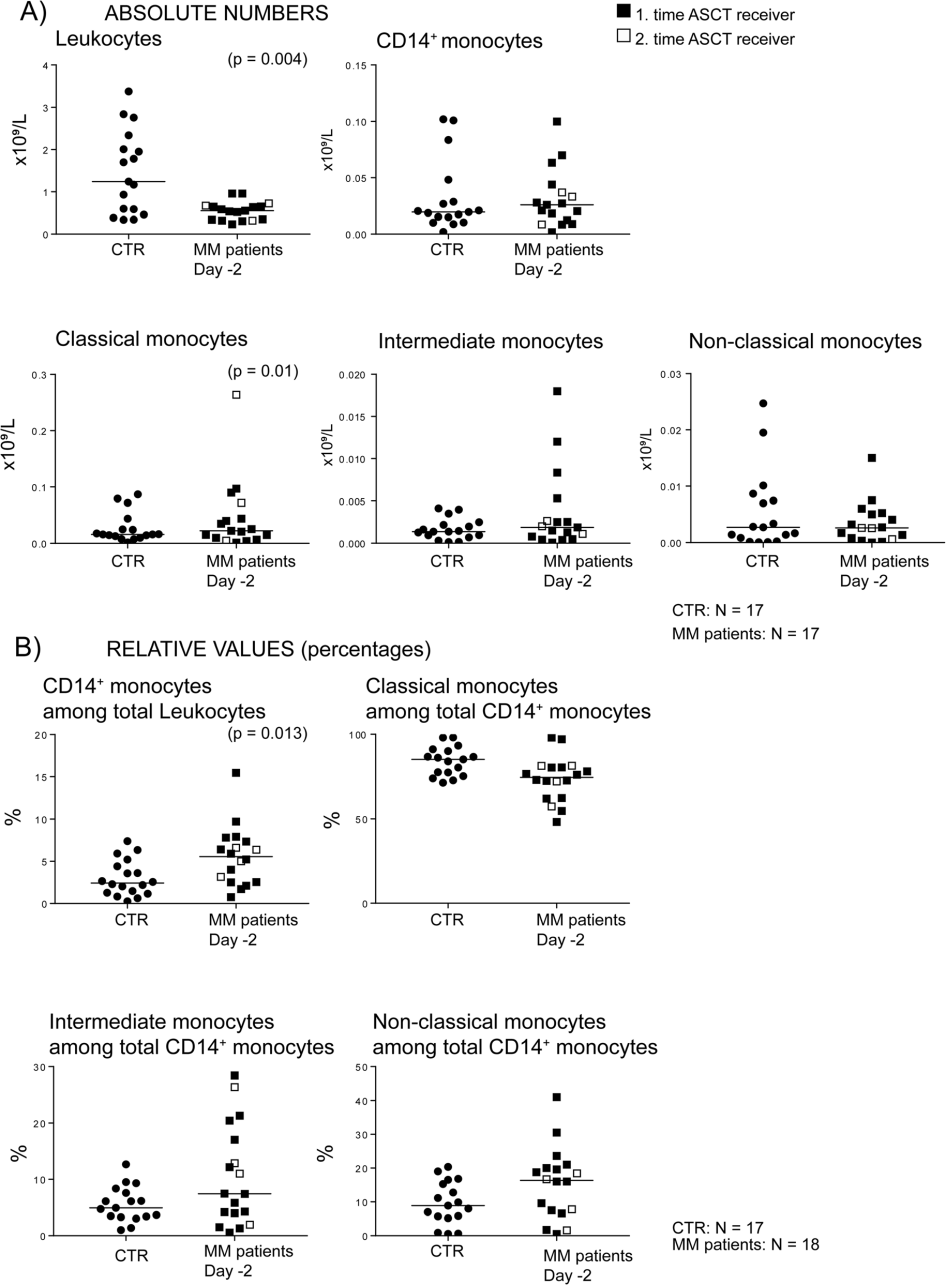
The peripheral blood levels of total leukocytes, total monocytes and monocyte subsets; a comparison between healthy controls and multiple myeloma patients examined after the initial induction chemotherapy and stem cell harvesting with cyclophosphamide plus G-CSF, i.e. immediately before conditioning high-dose melphalan therapy (pre-transplant day − 2). (**a**, UPPER FIGURES) We used flow cytometry to estimate the concentrations of total circulating leukocytes, total CD14^+^ monocytes and classical, intermediate and non-classical monocyte subset. The results for 17 patients (Table 1, patients 2–18) were compared with 17 healthy controls (CTR) individuals. Three of the 17 patients received their second auto-transplantation. (**b**, LOWER FIGURES) The percentage of circulating CD14^+^ monocytes among total leukocytes and the percentages of classical, intermediate and non-classical monocytes among total CD14^+^monocytes were estimated. The results for 18 patients (Table 1, patients 1–18) were compared with the 17 healthy individuals. Four patients received their second auto-transplantation. In all the figures, black symbols represent the levels for patients receiving their first auto-transplantation whereas open symbols represent levels for patients receiving their second transplantation. The Wilcoxon’s test for paired samples was used for statistical analyses and the *p*-values for statistically significant differences are indicated in the figure

We first compared the total leukocyte counts in peripheral blood for myeloma patients (Table 1, patients 2–18) and the healthy controls (12 males and 5 females, median age 51 years). The patients were tested immediately before high-dose melphalan conditioning, i.e. after initial induction treatment (see Table 1) followed by stem cell mobilization/collection based on cyclophosphamide plus G-CSF. At this time point they showed significantly decreased total leukocyte counts compared with the controls (Fig. 1a, *p* = 0.004), and patients receiving their first and second auto-transplantation showed a similar decrease. The decreased leukocyte counts were seen with both analytical methods (flow cytometry with counting beads, measurement by clinical hematology instrument), and the levels measured by these two methods were significantly correlated (Pearson correlation coefficient 0.963, *p*-value 0.0001). The total leukocyte levels prior to melphalan conditioning showed no association with age, induction treatment (regimen, number of cycles), response to induction treatment, circulating CD34^+^ cell level at the day of harvesting or duration of posttransplant neutropenia/cytopenia (data not shown).

**Table 1:** The characteristics of the myeloma patients included in the study

Patient	Age^d)^	M-component	Transplant^a)^	Conditioning therapy (drugs, number of cycles)		Effect of induction	CD34 count at the time of harvesting ^b)^	Duration of cytopenia^c)^	
				Pretransplant treatment	Number of cycles			Neutrophils	Thrombocytes
1	60–70	IgG MM	Second	CVD	4	VGPR		5	2
2	60–70	IgG MM		CVD	4	VGPR	171,270	4	1
3	60–70	IgA MM	Second	CVD	4	VGPR		5	3
4	50–60	IgG MM		CVD	5	VGPR	32,180	5	5
5	50–60	IgG MM		CVD	5	PR	48,635	2	1
6	30–40	LCD		CVD (1), KRD (4)	5	VGPR	70,570	5	3
7	60–70	IgG MM		VTD (1), CVD (2)	3	VGPR	218,945	4	2
8	60–70	IgG MM		CVD	4	VGPR	90,930	4	4
9	60–70	IgA MM		CVD	5	PR	42,285	3	3
10	50–60	IgG MM	Second	PVD	6	PR		5	4
11	50–60	IgA MM		CVD	4	VGPR	32,125	5	4
12	60–70	IgA MM	Second	VRD	4	VGPR		5	2
13	60–70	LCD-L		CVD	4	VGPR	60,410	5	4
14	60–70	IgG MM		CVD, VTD, VD (3)	5	VGPR	123,190	6	1
15	60–70	IgG MM		VCD	4	PR	37,060	8	5
16	60–70	LCD-K		RD	4	VGPR	36,010	4	6
17	50–60	IgA-MM		CVD	4	VGPR	132,000	4	5
18	60–70	LCD-L		CVD (4 before and 2 after harvesting)	6	VGPR	27,690	5	5
19	50–60	IgG MM	Second	CVD	4	VGPR		5	3
20	50–60	Amyloid-MM IgG-L		CVD (3), VTD (1)	4		185,800	4	2
21	40–50	IgG MM		CVD (4 before harvesting), VTD (2 after), RD (2 after)	8	VGPR	56,380	4	6
22	50–60	LCD-L	Second	CVD	4	PR		7	5
23	50–60	IgG MM	Second	CVD, VTD, VD (4)	6	PR		5	5
24	50–60	LCD-L		CVD	4	VGPR	74,800	3	4
25	60–70	LCD-K	Second	CVD	4	PR		3	2

*Abbreviations*: *CVD* Cyclophosphamide, bortezomib (Velcade®), dexamethasone, *LCD* Light chain disease type lambda (L) or kappa (K), *MM* Multiple myeloma, *PR* Partial response, *RD* Lenalidomide (Revlemide®) plus dexamethasone, *VGPR* Very good partial response, *VRD* Bortezomib, lenalidomide (Revlemide®), dexamethasone, *VTD* Bortezomib (Velcade®), thalidomide, dexamethasone

^a)^ Patients undergoing their second auto-transplantation are indicated; the stem cell graft was the same as for the first transplantation for all these patients

^b)^ The peripheral blood concentration of CD34^+^ cells on the (first) day of harvesting is given; the level is expressed as × 10^3^ cells/mL

^c)^ Neutropenia was defined as the time from the first day of neutrophil peripheral blood concentration ≤ 0.2 × 10^9^/L until the first of three consecutive days with neutrophils exceeding 0.2 × 10^9^/L or alternatively the first day with neutrophil counts > 10 × 10^9^/L. The duration of thrombocytopenia was defined as the number of days from the first day of peripheral blood thrombocyte counts below 20 × 10^9^/L until the first day with thrombocyte count above 20 × 10^9^/L without thrombocyte transfusion

^d)^ The age of patients are grouped

### Myeloma patients show normal peripheral blood concentrations of total monocytes but decreased levels of classical monocytes prior to high-dose melphalan

The preconditioning peripheral blood concentrations of total CD14^+^ monocytes did not differ between the 17 myeloma patients (Table 1, patients 2–18) and 17 healthy controls (Fig. 1a). However, classical monocyte concentrations were then slightly increased (Fig. 1a, *p* = 0.01) whereas we could not detect any significant differences between patients and controls for intermediate and non-classical monocytes. The three patients admitted for their second auto-transplantation showed total monocyte and monocyte subset concentrations within the range for the patients admitted for their first transplantation (Fig. 1). Thus, the effect of mobilization/conditioning on circulating monocytes is a non-random effect mainly affecting the classical monocyte subset.

The total monocyte concentrations prior to the conditioning therapy showed no association with age, induction treatment (regimen, number of cycles), response to induction treatment, circulating CD34^+^ cell level at the first day of harvesting or the duration of posttransplant neutropenia/cytopenia (data not shown). The same was true for classical, intermediate and non-classical monocytes except that pre-harvesting CD34^+^ cell levels showed significant correlations to absolute and relative levels of intermediate (*r* = 0.78/*p* = 0.001 and *r* = 0.75/*p* = 0.002, respectively) and non-classical monocytes (*r* = 0.63/*p* = 0.017 and *r* = 0.61/*p* = 0.047, respectively.

### Myeloma patients are heterogeneous with regard to the preconditioning monocyte subset distribution/percentage in peripheral blood

We first compared the relative levels of circulating total CD14^+^ monocytes (percentage of total leukocytes) and the various monocyte subsets (percentage of total monocytes) for 18 newly diagnosed myeloma patients (Table 1, patients 1–18) and the 17 healthy controls. The preconditioning percentage of CD14^+^ monocytes among total leukocytes was increased for the patients; this was expected since total leukocyte levels were decreased whereas the monocyte concentration was not significantly altered before melphalan conditioning (Fig. 1b, *p* = 0.013).

The preconditioning percentages of the classical, intermediate and non-classical monocyte subsets among CD14^+^ monocytes did not differ between patients and healthy controls (Fig. 1b). However, the variation range was wider for the patients both for the percentage of total CD14^+^ monocytes and the three monocyte subsets. Firstly, classical monocytes constituted a majority of the CD14^+^ monocytes (corresponding to > 70%) both for the healthy controls and for all except five patients. We also observed wide variation ranges for the intermediate and non-classical monocyte subsets; exceptional patients showed intermediate monocyte levels exceeding 15% and non-classical monocytes levels up to 40% of the total CD14^+^ monocytes (Fig. 1b). Wide variations were observed both for patients admitted to their first auto-transplantation and for the four patients admitted to their second transplantation. The percentages of total monocytes and monocyte subsets showed no significant associations with age, induction treatment, response to induction treatment, CD34^+^ cell level at the day of harvesting or time until posttransplant neutrophil/platelet reconstitution (data not shown).

### The concentrations of circulating CD14^+^ monocytes decrease early after melphalan conditioning

**Fig. 2:**
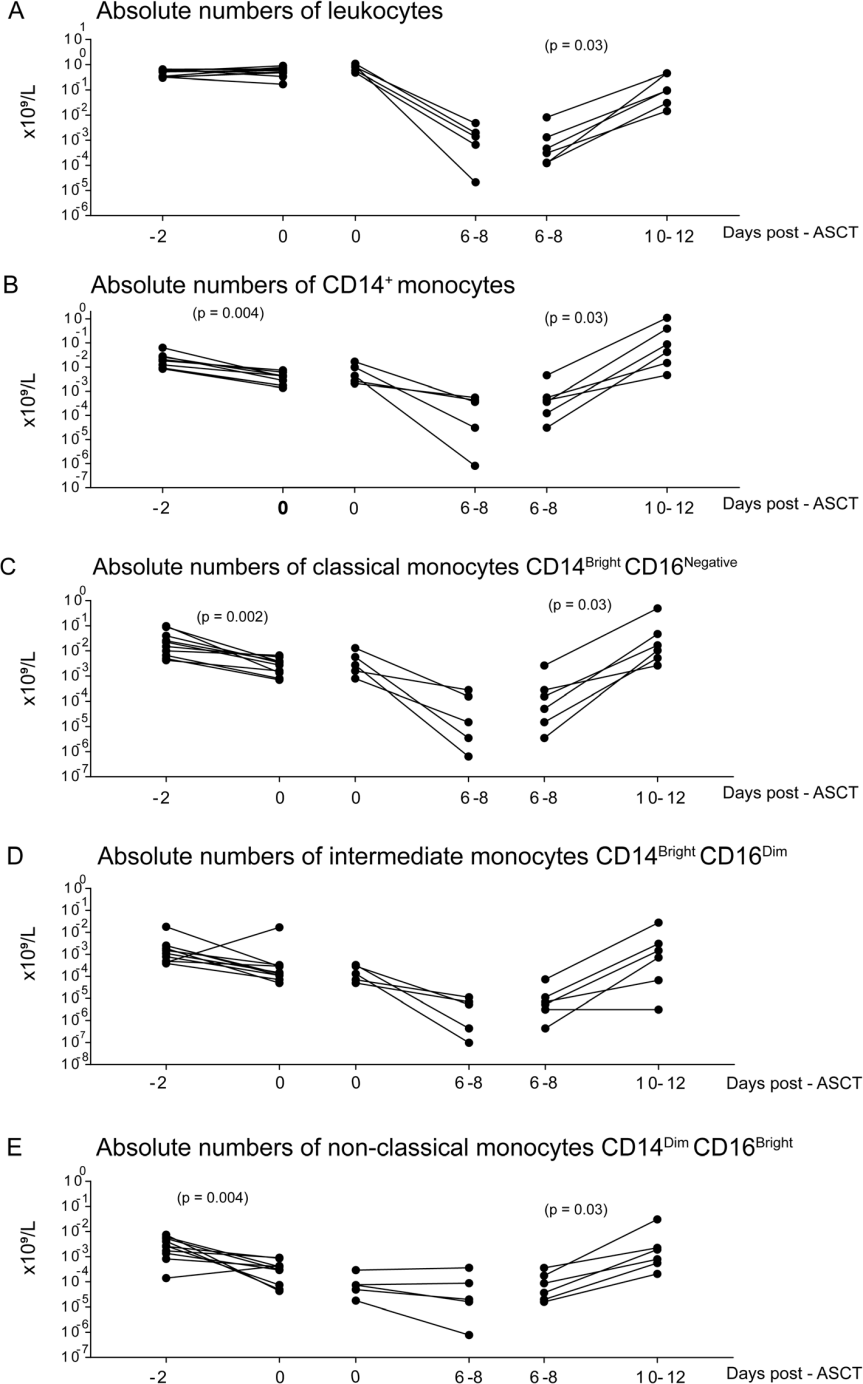
The peripheral blood concentrations of total leukocytes (**a**), CD14^+^ monocytes (**b**), classical (**c**), intermediate (**d**) and non-classical (**e**) monocytes in auto-transplanted myeloma patients. We estimated the cell concentrations by flow cytometry. The left parts of each panel show a comparison of peripheral blood levels before versus 2 days after the conditioning melphalan therapy (day − 2 versus day 0; Table 1, patients 7–9, 12–18). The middle parts show a comparison of levels at the time of transplantation (day 0) versus the levels during the period of severe posttransplant melphalan-induced pancytopenia with neutrophils < 0.2 × 10^9^/L and thrombocyte transfusion dependency (tested 6–8 days posttransplant; Table 1, patients 14, 15, 17, 22, 24). The right panel show the comparison of peripheral blood levels during the severe pancytopenia versus the levels during initial hematopoietic and immunological reconstitution (tested 10–12 days posttransplant with increasing neutrophils > 0.2 × 10^9^/L and thrombocyte transfusion independence; Table 1 patients 15, 17, 18, 22–24). The Wilcoxon’s test for paired samples was used for statistical analyses and significant p-values are indicated in the figure (ASCT, autologous stem cell transplantation)

We compared the peripheral blood levels of total leukocytes and CD14^+^ monocytes before the conditioning therapy (day − 2) and 2 days later immediately before the autologous stem cell reinfusion (day 0). Ten patients were available for this paired comparison (Table 1, patients 7–9, 11–17), and total leukocyte levels were not altered 2 days after the melphalan infusion (i.e. immediately before transplantation, see Fig. 2a). In contrast, the concentrations of circulating monocytes were significantly decreased 2 days after the conditioning, and a similar decrease was observed when total monocyte levels were analyzed by clinical hematology instrument(data not shown) and when using whole-blood staining for flow cytometric analysis of CD14^+^ monocytes (Fig. 2b). A comparable decrease was seen for patients receiving their first and the second auto-transplantation, but neutrophil levels were increased for many patients (see below) so that total leukocyte levels were not significantly altered.

We finally compared the levels of circulating neutrophils, total lymphocytes and thrombocytes (estimated by clinical hematology instrument) immediately before and 2 days after melphalan conditioning (Table 1, patients 7–9, 11–17). The neutrophil levels 2 days after melphalan were slightly increased (median level 3.6 versus 4.9 × 10^9^/L, *p* = 0.06), whereas lymphocyte (median level 1.0 versus 0.2 × 10^9^/L, *p* = 0.002) and thrombocyte levels (median levels 225 versus 177 × 10^9^/L, p = 0.002) were significantly decreased similar to the monocyte levels.

### Melphalan conditioning causes an early decrease in the percentages of circulating classical and non-classical monocytes whereas the levels of the intermediate subset are not altered

We compared the peripheral blood percentages of classical, intermediate and non-classical monocytes at day − 2 pre-transplant (i.e. before conditioning) and day 0 (i.e. before stem cell transplantation) for 10 patients (Fig. 2c-e; Table 1 patients 7–9, 11–17). The concentrations of all three monocyte subsets were decreased for most patients 2 days after the conditioning, but the difference reached statistical significance only for the classical (Wilcoxon’s test for paired samples; *p* = 0.002) and non-classical subsets (*p* = 0.0039). Thus, melphalan conditioning has a nonrandom early effect on circulating monocyte subsets. However, we observed a wide variation in the percentage of the various monocyte subsets among total CD14^+^ monocytes in preconditioning samples (Fig. 1), and wide variations persisted after conditioning both for the classical (variation range 41–93%), intermediate (2–48%) and non-classical (2–51%) monocyte subsets.

### The early effect of melphalan conditioning on monocyte concentrations differs between patients receiving induction treatment with and without immunomodulatory drugs

IMiDs can alter the monocyte phenotype [24], and we therefore compared the effect of the melphalan conditioning for patients receiving induction treatment with or without IMiDs (i.e. thalidomide, lenalidomide, pomalidomide). A total of 13 patients were included in this analysis. At day 0 (the day of transplantation) patients receiving induction treatment including IMiDs showed decreased concentrations of circulating total monocytes (median level 0.0023 × 10^9^/L with range 0.001–0.01 versus 0.0061 × 10^9^/L with range 0.003–0.03; *p* = 0.045) and classical monocytes (median 0.0022 × 10^9^/L with range 0.001–0.010 versus 0.0057 × 10^9^/L with range 0.0003–0.016; *p* = 0.046) compared with patients receiving induction treatment without these drugs). Furthermore, the absolute and relative levels of total monocytes and the various monocyte subsets 2 days after the conditioning therapy (i.e. on day 0, the day of transplantation) showed no association with age, response to induction treatment or levels of circulating CD34^+^ cells at the first day of harvesting (data not shown). Finally, the day 0 pretransplant levels of circulating neutrophils, total lymphocytes and thrombocytes did not differ between patients that had received induction therapy with and without IMiDs (data not shown). Thus, the IMiDs seem to have a non-random effect on the various monocyte subsets that becomes detectable after the melphalan infusion.

### The peripheral blood concentrations of all three monocyte subsets show a further decrease during the period of severe neutropenia

We investigated the peripheral blood concentrations of the three monocyte subsets during the period of severe neutropenia for 8 myeloma patients (Table 1 patients 13, 14, 16, 17, 21–24); for five of these patients we could compare the levels immediately before stem cell reinfusion with the levels during severe cytopenia (patients 13, 14, 16, 17, 21). As expected the concentrations of all three monocyte subsets, especially the classical and intermediate subsets, decreased to low levels during cytopenia (Fig. 2). In contrast, the relative levels (i.e. percentage among total CD14^+^ monocytes) varied during pancytopenia when tested 6–8 days after stem cell reinfusion. All patients showed < 5% intermediate monocytes, whereas classical monocyte levels varied between 8 and 92% (median 62%) and non-classical monocytes also showed a considerable variation (median 18%, range 3–57%).

### Auto-transplanted myeloma patients show expected early hematological reconstitution

The levels of circulating total leukocytes, neutrophils and thrombocytes were measured by clinical hematology instrument) for all our patients. Neutrophil reconstitution was defined as the first of 3 days with neutrophils above 0.2 × 10^9^/L. The median time from first day of neutropenia (i.e. first day with neutrophils ≤0.2 × 10^9^/L) until neutrophil reconstitution was 4 days (range 2–9 days). Furthermore, thrombocyte reconstitution was defined as the first out of three consecutive days with thrombocyte counts above 20 × 10^9^/L in transfusion-independent patients. The median duration of thrombocytopenia (i.e. thrombocyte levels below 20 × 10^9^/L) was 4 days (range 1–6 days). Finally, time to neutrophil/thrombocyte reconstitution did not differ between patients receiving their first or second auto-transplantation and showed no significant associations with preconditioning (i.e. day − 2) or pre-transplant (i.e. day 0) total monocyte levels.

### Auto-transplanted myeloma patients show early monocyte reconstitution

The absolute levels of total monocytes were followed daily during the period of early hematological reconstitution for 24 consecutive patients. The median time from transplantation until the monocyte levels exceeded the lower normal limit (0.04 × 10^9^/L) was 10 days; the median monocyte level was then 0.23 × 10^9^/L (range 0.05–0.78 × 10^9^/L). The neutrophil levels at the first day of monocyte normalization were generally below the lower normal limit (median 0.5 × 10^9^/L, range 0.1–3.8 × 10^9^/L), i.e. for 18 patients the neutrophil levels were still below the lower normal limit. All patients still had severe thrombocytopenia (median 29 × 10^9^/L, range 13–38 × 10^9^/L) at the first day of monocyte normalization. Finally, there was no significant association between preconditioning or pre-transplant total monocyte levels and time to normalized circulating monocyte levels, and monocyte normalization did not differ for patients receiving induction treatment with or without IMiDs (data not shown).

**Fig. 3:**
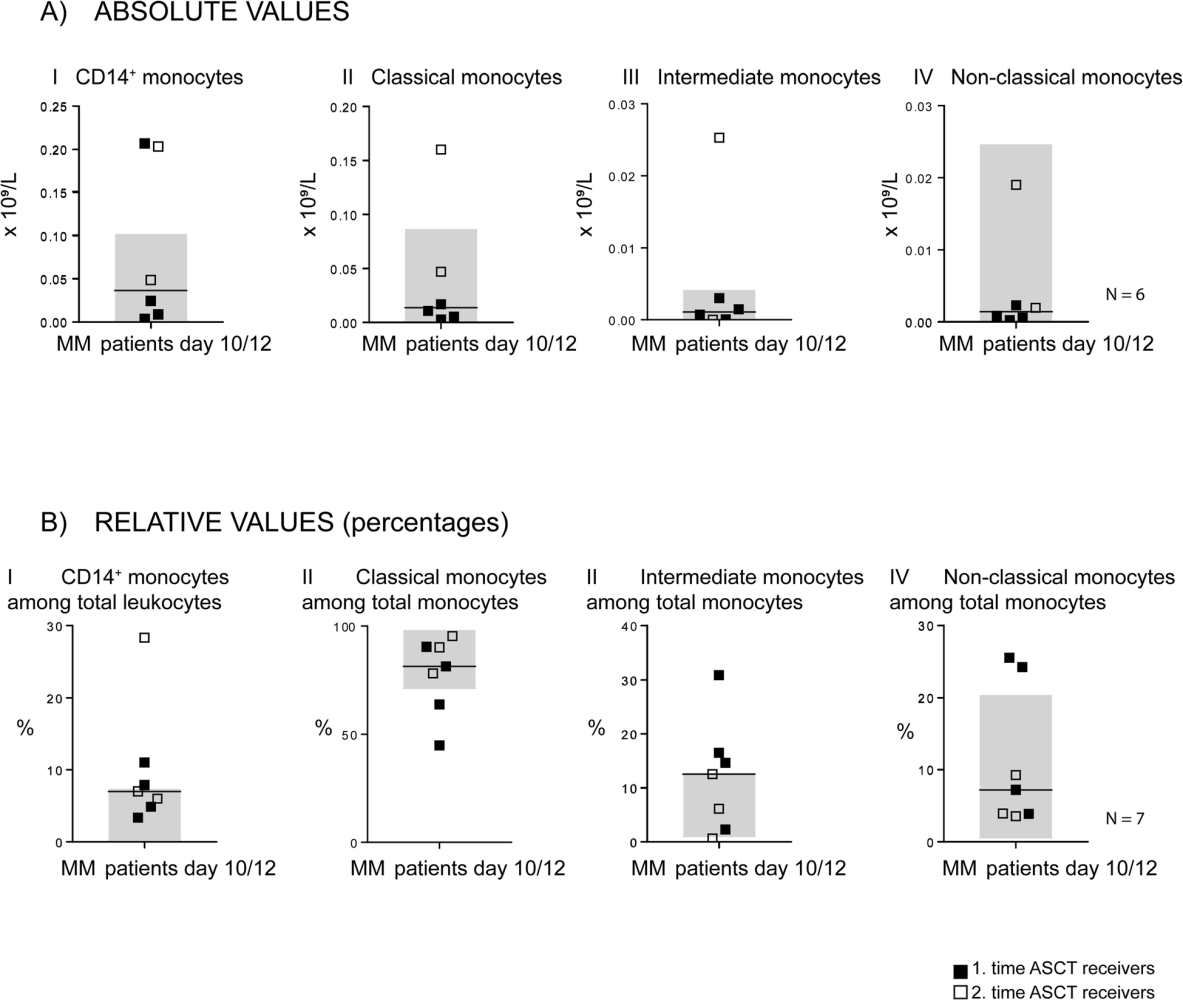
The peripheral blood concentrations (**a**, upper part, *n* = 6) and percentages (**b**, lower part, *n* = 7) of total CD14^+^ monocytes and classical, intermediate and non-classical monocyte subsets in auto-transplanted myeloma patients. Monocyte levels were estimated by flow cytometry. We investigated the levels for a total of eight patients (Table 1, patients 15, 17, 18, 22–24) during initial hematopoietic and immunological reconstitution (tested 10–12 days posttransplant) when the patients showed increasing neutrophils > 0.2 × 10^9^/L and thrombocyte transfusion independence). Horizontal lines indicate median values after 10–12 days. The shaded parts of each figure indicate the variation range of the corresponding peripheral blood levels tested 2 days pre-transplant (referred to as day − 2), i.e. immediately before high-dose melphalan conditioning therapy. Black symbols represent patients receiving their first auto-transplant, open symbols represent patients receiving their second auto-transplantation

We compared the absolute and relative levels of various monocyte subsets at day + 10/+ 12 posttransplant with the corresponding preconditioning levels (day − 2); paired samples were then available only for eight patients (Fig. 3; Table 1 patients 14, 16, 17, 21–25). This posttransplant time point corresponds to the initial neutrophil reconstitution, but the neutrophil levels were still below the lower normal limit for six of the eight patients (median level 0.7 × 10^9^/L, range 0.2–6.8 × 10^9^/L). The thrombocyte counts for all patients (median 30 × 10^9^/L, range 20–52 × 10^9^/L) were also below the lower normal limit. However, even at this early time point only 10–12 days post-transplant most patients showed normalized absolute (concentration) and relative (percentage) levels of total CD14^+^ monocytes as well as the three monocyte subsets within the pre-transplant variation range.

One of our collaborating local hospitals only investigated peripheral blood neutrophil but not monocyte counts at the out-patient evaluations; for this reason peripheral blood monocyte counts were only available at later time points for 15 patients. The total monocyte count (normal range 0.04–1.30 × 10^9^/l) was tested early after neutrophil and platelet reconstitution, and at this time point (median time 14 days posttransplant, range 14–16) nine patients showed normal and six patients showed increased counts (median 0.95 × 10^9^/L, range 0.40–2.1 × 10^9^/l). The total monocyte counts tested at a later time point (median 30 days posttransplant, range 27–39 days) showed increased levels for a minority of four patients (median level 0.97 × 10^9^/L, range 0.20–1.78 × 10^9^/L).

### Most patients shows disease stabilization lasting at least 2 years after the first transplantation

The posttransplant observation time for patients receiving their first auto-transplantation was 27–36 months (median 32 months). Five patients had disease progression less than 2 years post-transplant, one patient was lost from follow-up and all other patients remained in plateau phase during follow-up. Progression-free survival less than 2 years showed no association with precondition (*n* = 16) or pretransplant (*n* = 18) total monocyte or monocyte subset levels or with posttransplant time to normalized total monocyte levels (*n* = 24) (data not shown).

## Discussion

Autologous stem cell transplantation is widely used in the treatment of younger myeloma patients up to 70 years of age [25]. The pre-transplant conditioning therapy has direct anti-leukemic effects, but previous studies suggest that immune-mediated anti-myeloma activity may also contribute to the effect of this therapy [26–28]. The lymphoid reconstitution has been investigated in previous studies [29], but the monocytes are less well characterized and for many of the previous studies the induction treatment included conventional cytotoxic drugs and not proteasome inhibitors or IMiDs. In our present study, we investigated the levels of circulating total monocytes and monocyte subsets in auto-transplanted myeloma patients. Although our study is relatively small, we observed that the pre-transplant induction and stem cell mobilization by cyclophosphamide plus G-CSF seemed to have only a minor effect on the preconditioning monocyte levels even though the concentration of total circulating leukocytes was decreased compared with the healthy controls.

Our present observations will probably not only depend on the use of IMiDs and proteasome inhibitors in the induction treatment but on the overall clinical and biological context of our patients. One should emphasize that the use of cyclophosphamide in stem cell mobilization will probably influence our results. The same may be true for our use of posttransplant G-CSF therapy, e.g. through its effects on systemic metabolic regulation that influence the metabolic environment of regenerating hematopoietic and immunocompetent cells [30]. Furthermore, studies in healthy donors show that G-CSF has a mobilizing effect on many different immunocompetent cells, including monocytes [31, 32]. These effects differ between healthy donors; they will also influence the levels of immunocompetent cells in the stem cell grafts and possibly also outcome in allotransplant recipients. To the best of our knowledge it is not known whether similar differences exist for auto-transplanted myeloma patients, and unfortunately we do not have information about graft levels of various monocyte subsets for our patients.

We analyzed the total number of monocytes by two different methodological approaches, i.e. by using a hemocytometer and by using flow cytometry to estimate the levels of CD14^+^ total monocytes. Both these analyses showed that the preconditioning patient levels did not differ from healthy controls, whereas the levels 2 days after the conditioning (i.e. immediately before stem cell reinfusion) were decreased compared with the preconditioning levels. However, the levels of CD14^+^ monocytes were lower than the monocyte levels estimated in the alternative assays, and this difference is probably due to a random loss of cells during the washing steps.

Our studies included all except one patient from a defined geographic area and during a defined time period; for this reason it should be regarded as a population-based study. We could not investigate all patients at every time point during the treatment. However, we would emphasize that this was due to practical reasons such as transfer of patients to their local hospital or long traveling distance from their home to the transplantation center; it was not because of the disease, the treatment or development of complications. Leukocyte levels show diurnal variations [33–36], and for this reason we sampled the patients only in the morning, and shipment of samples or analysis of cryopreserved cells was not possible due to our standardized methods for handling of the samples [37].

Our present study showed that the preconditioning patient levels did not differ from healthy controls, i.e. the myeloma disease itself, the induction treatment and the stem cell mobilization by cyclophosphamide plus G-CSF have only minor effects on monocytes except for a slight increase of classical monocytes. In contrast, the melphalan conditioning seemed to have a nonrandom effect of the monocyte subsets before an early reconstitution of all three subsets was observed. However, it should be emphasized that there is a wide variation between patients with regard to the effects of the conditioning therapy. A short duration of this monocytopenia is also suggested by previous studies [7, 8], but our study is the first to suggest that this is true also for patients receiving IMiD- or proteasome inhibitor-based induction therapy and for different monocyte subsets. Furthermore, the studies by Callander et al. [38] suggest that even though monocyte levels are normalized at day 100 posttransplant, the levels of total CD14^+^ and CD14^+^CD16^low/negative^ classical monocytes are then associated with prolonged progression-free survival after auto-transplantation. Thus, taken together these studies show that monocyte reconstitution occurs early (according to our study very early) after auto-transplantation, but despite this normalization there is still a relatively wide variation between patients and this heterogeneity in monocyte (subset) levels seems to persist until day 100 posttransplant and may even have a prognostic impact. The antimyeloma effect of posttransplant monocyte targeting may therefore vary between patients and depend on the monocyte subset profile. IL6 is regarded as a possible target in multiple myeloma [39]; monocytes constitute a subset of the bone marrow stromal cells that are regarded as important regulators of both normal and malignant hematopoietic cells [16, 40]. IL6 is released by monocytes, especially classical monocytes, in response to ligation of various Toll-like receptors, and therapeutic targeting of IL6/monocytes may therefore be most effective for those patients with high levels of classical monocytes.

Most of our patients received only 3 or 4 induction cycles before stem cell transplantation, whereas 6 cycles are now often recommended, especially for patients who have not received a complete remission [25, 41, 42]. Alternative induction cycles have also been used in other studies [33, 41, 42], and future studies have to clarify whether our present results are representative also for patients receiving additional cycles or alternative induction treatment.

Our comparison of induction treatments with and without IMiDs suggests that the post-conditioning monocyte concentrations are influenced by the previous use of immunomodulatory drugs in the induction therapy, whereas the capacity of stem cell mobilization and response to the induction therapy are less important. However, G-CSF responsiveness (i.e. CD34^+^ cell mobilization) was associated with the levels of intermediate and non-classical monocytes before conditioning therapy.

Previous studies have shown that early posttransplant lymphoid reconstitution is associated with a favorable prognosis of auto-transplanted myeloma patients [26–29]. More recent studies suggest that this effect may be due to early NK cell reconstitution [29]. Even though monocytes have important immunoregulatory functions, the previous studies have not investigated whether the early monocytic reconstitution is required for the prognostic impact of early lymphoid reconstitution. A recent study of allotransplant recipients suggests that monocytes can mediate anti-myeloma effects [43], and monocytes derived from auto-transplanted patients may even be used for immunotherapy due to their presentation of myeloma-associated peptides to the adaptive immune system [44]. Our present studies thus suggest that a close to normal monocyte system is present in myeloma patients even early after auto-transplantation and may then be an immunotherapeutic target.

Monocytes and macrophages are important members of the bone marrow stem cell niches that support both normal and malignant hematopoiesis [40]. IMiDs can alter the differentiation of monocytes [24], and we therefore investigated whether the use of such drugs for induction therapy was associated with an altered balance between monocyte subsets later during treatment or with other differences in hematopoietic reconstitution between patients. Our present study showed that the type of induction therapy actually has an influence on monocytes/monocyte subsets, but this difference was only detected after additional melphalan therapy in pre-transplantation (Day 0) samples.

The observation time for our patients was relatively short (27–35 months) for our patients that received their first auto-transplantation and a majority of them were still in a plateau phase. We could not detect any associations between time to progression (i.e. progression before 2 years posttransplant) and monocyte subset levels/reconstitution. However, these data should be interpreted with great care because the patient cohort is relatively small for such analyses and the observation time is short and patients with early relapse are few.

## Conclusions

Although our study is relatively small, we observed that the total level of circulating monocytes is normalized early after auto-transplantation for multiple myeloma. However, the levels of various monocyte subsets show considerable variation between patients. Clinical studies including larger number of patients and a longer observation time are needed to clarify whether these differences are associated with overall survival, time to relapse and/or frequencies of severe infections.

## Methods

### Aim, design, characteristics of myeloma patients and healthy controls

Proteasomal inhibitors and IMiDs are now commonly used in induction treatment of young and fit myeloma patients prior to stem cell harvesting and auto-transplantation. These drugs may thereby influence the pretransplant immunological status of the patients and the immunocompetent cells in the stem cell graft. The aim of our present study was therefore to investigate the preconditioning status of the monocyte system in myeloma patients treated with induction chemotherapy based on IMiDs or proteasomal inhibitors, and to characterize the monocyte subset levels in autotransplanted patients during the early posttransplant period until hematological reconstitution.

Our hospital is the only center for stem cell transplantation in a defined geographical area of Norway (Health Region III), and our patients represent all myeloma patients except one receiving autologous stem cell transplantation in this area during an 8 months period. Our study should therefore be regarded as a population-based study of unselected patients.

The diagnosis of multiple myeloma was based on generally accepted criteria [1, 45], and induction therapy was initiated in accordance with generally accepted international guidelines [45, 46]. The patient characteristics are presented in Table 1. All patients received premobilization therapy including either a proteasome inhibitor or an IMiD; this induction treatment was followed by stem cell mobilization using cyclophosphamide plus G-CSF [47, 48]. The stem cell grafts were cryopreserved in 5% dimethyl sulfoxide and stored in liquid nitrogen until reinfusion [49, 50]. All patients were transplanted with at least 6 × 10^6^ CD34^+^ cells per kilo body weight, and the median time from start of induction to transplantation was 16 weeks (range 13–28 weeks). For patients receiving their first auto-transplantation the grafts had been stored for 3–5 weeks, whereas for those patients receiving their second transplantation the grafts were stored for at least 2 years. All patients received conditioning therapy with melphalan (Fresenius Kabi, Oslo, Norway), 200 mg/m^2^ administered as an intravenous infusion 2 days before stem cell reinfusion. They received G-CSF 5 μg/kg from day + 4 posttransplant until stable neutrophil recovery, i.e. peripheral blood neutrophil levels above 0.2 × 10^9^/L for three consecutive days or exceeding 10 × 10^9^/L.

The normal controls were healthy blood donors; in accordance with the approved routines at the Blood Bank, Haukeland University Hospital peripheral venous blood samples for medical research were donated after written informed consent.

### Blood sampling

Blood samples were drawn in ACD-A (9 mL, #248368, BD Vacutainer, San Jose, CA, USA) blood sampling vacuum tubes. We collected the first patient sample immediately before melphalan conditioning (day − 2). The second sample was collected 2 days later immediately before reinfusion of the peripheral blood stem cell graft (day 0). The third sample was collected on day + 6 posttransplant when patients had severe neutropenia (peripheral blood neutrophil counts below < 0.2 × 10^9^/L) and thrombocytopenia. The last sample was collected on the first or second day with peripheral blood neutrophil counts exceeding 0.2 × 10^9^/L (10–12 days posttransplant). The control samples were derived from 17 healthy blood donors (5 females and 12 males, median age 51 years with range 22–82 years). All samples were processed at room temperature within 120 min. All samples were collected between 08:00 and 10:00 am. It was not possible to get samples from all patients at all four time points; this was due to either transfer to their local hospitals after stem cell reinfusion or the patient was not available for sampling at the indicated time in the morning.

### Flow cytometric analysis

Four mL of ACD-A (9, #248368, BD Vacutainer) anticoagulated whole blood and 46 mL of lysing buffer (# 55589, BD Biosciences) were mixed and incubated for 15 min at room temperature. Subsequently, leukocytes were collected by centrifugation (400×g, 5 min, room temperature) and thereafter washed in phosphate-buffered saline with 1% Bovine Serum Albumin (BSA, Bovine Serum Albumin Fraction V #10735086001, Sigma-Aldrich/Merc KGaA, Darmstadt, Germany),. The cells were reconstituted in 200 μL 1% BSA/PBS with 10% immunoglobulin solution (Octagam 100 mg/mL, Octapharma, Lachen, Switzerland). The following mouse anti-human antibodies were included in the antibody panel (all from BD Biosciences, San Jose, CA, US): CD14 Alexa 488 (Clone M5E2), CD56 Alexa 647 (Clone B159), CD16 PerCpCy™5–5 (Clone 3G8), CD45 V500 (Clone HI30), CD11b V540 (Clone ICRF44 (44)) and HLA-DR PE (Clone G46–6). The staining procedure and gating strategy for identification of monocytes and monocyte subsets has been described in detail in a previous methodological article [37].

All samples were analyzed by a 10-parameter BD FACS Verse flow cytometer equipped with 404, 488 and 640 nm lasers. We used BD FACSuite™ CS&T Research Beads (#650621, BD Biosciences, San Jose, CA, USA) for regular quality control of the instrument, single-stained compensation bead samples (#552843, BD Biosciences) for compensation and unstained samples as gating controls. At least 5000 monocytes were analyzed for each sample (based on SSC/FSC properties). We used counting beads (Count Bright Absolute Counting beads™, # C36950, Invitrogen™, Thermo Fischer Scientific, Waltham, MA USA) when estimating the concentrations of monocytes/monocyte subsets. FlowJo software (Tree Star, Inc., OR, USA) was used for analysis of the results.

### Analysis of total leukocytes, neutrophils, monocytes and thrombocytes in peripheral blood

Analyses of peripheral blood levels of total leukocytes and total monocytes were performed by using accredited clinical hematology instrument (Laboratory for Clinical Biochemistry and Hematology at Haukeland University Hospital).

### Statistical analyses

We applied IBM SSP statistics 23 for all statistical analyses. The Wilcoxon’s rank sum test and the Wilcoxon’s test for paired samples were used for comparison of different groups and for comparison of paired observations, respectively. The Pearson’s test was used for correlation analyses. Differences were regarded as statistically significant when *p*-values were below 0.05.

## Data Availability

The data sets used and analyzed during the study are available from the corresponding author on request.

## Abbreviation

IMiDs: Immune modulatory drugs
